# *Neisseria meningitidis *rifampicin resistant strains: analysis of protein differentially expressed

**DOI:** 10.1186/1471-2180-10-246

**Published:** 2010-09-24

**Authors:** Arianna Neri, Giuseppina Mignogna, Cecilia Fazio, Alessandra Giorgi, Maria Eugenia Schininà, Paola Stefanelli

**Affiliations:** 1 Department of Infectious, Parasitic and Immune-mediated Diseases, Istituto Superiore di Sanità, Viale Regina Elena 299, 00161 Rome, Italy; 2 Department of Biochemical Sciences, "A. Rossi Fanelli", University "Sapienza", Piazzale Aldo Moro 5, 00185 Rome, Italy

## Abstract

**Background:**

Several mutations have been described as responsible for rifampicin resistance in *Neisseria meningitidis*. However, the intriguing question on why these strains are so rare remains open. The aim of this study was to investigate the protein content and to identify differential expression in specific proteins in two rifampicin resistant and one susceptible meningococci using two-dimensional electrophoresis (2-DE) combined with mass spectrometry.

**Results:**

In our experimental conditions, able to resolve soluble proteins with an isoelectric point between 4 and 7, twenty-three proteins have been found differentially expressed in the two resistant strains compared to the susceptible. Some of them, involved in the main metabolic pathways, showed an increased expression, mainly in the catabolism of pyruvate and in the tricarboxylic acid cycle. A decreased expression of proteins belonging to gene regulation and to those involved in the folding of polypeptides has also been observed. 2-DE analysis showed the presence of four proteins displaying a shift in their isoelectric point in both resistant strains, confirmed by the presence of amino acid changes in the sequence analysis, absent in the susceptible.

**Conclusions:**

The analysis of differentially expressed proteins suggests that an intricate series of events occurs in *N. meningitidis *rifampicin resistant strains and the results here reported may be considered a starting point in understanding their decreased invasion capacity. In fact, they support the hypothesis that the presence of more than one protein differentially expressed, having a role in the metabolism of the meningococcus, influences its ability to infect and to spread in the population. Different reports have described and discussed how a drug resistant pathogen shows a high biological cost for survival and that may also explain why, for some pathogens, the rate of resistant organisms is relatively low considering the widespread use of a particular drug. This seems the case of rifampicin resistant meningococci.

## Background

Management of meningococcal disease requires immediate treatment of patients and chemoprophylaxis of contacts. For the latter, rifampicin is the most frequently used antibiotic. However, although it has been utilized routinely worldwide for more than 30 years, few cases of rifampicin resistant meningococci have been reported [1]. This scarce diffusion is intriguing and the reduced virulence of these strains in terms of the bacterium's survival in the bloodstream of mice, as shown in an *in vivo *model, suggests a major biological cost for the microorganism [2].

The resistance phenotype is correlated with a set of mutations in the *rpoB *gene, encoding the β subunit of RNA polymerase, resulting in amino acid substitutions at one of the following codons: Asp542, Ser548, His552, Ser557, Gly560 [3-6]. Moreover, other mechanisms have been described in both *Neisseria meningitidis *and in *Neisseria gonorrhoeae *[7,8], i.e. resistance to diverse hydrophobic agents, including Triton X, is associated with mutations in the *mtrR *gene and in its promoter [7,9,10]. Overall, in other species, such as *Mycobacterium tuberculosis*, resistance was not related to any changes in the *rpoB *gene in around 5% of clinical rifampicin resistant isolates [11]. Rifampicin binds to DNA-dependent RNA polymerase and inhibits initiation of RNA synthesis which is not a mechanism of action shared with other antibiotics. This effect on RNA polymerase appears to result from drug binding in the polymerase subunit deep within the DNA/RNA channel where direct blocking of the elongating RNA can occur.

Little is known of the protein expression of *N. meningitidis *resistant to rifampicin and how this contributes to pathogenesis. In the present study, soluble proteins of two rifampicin resistant and one susceptible meningococci isolated in Italy, and previously described [5], were analysed by two-dimensional electrophoresis (2-DE) combined with mass spectrometry (MALDI-ToF). The method has been chosen because it is a comprehensive approach to investigate the protein content of a pathogen [12], and in this context helpful to identify differential expression in specific proteins in particular in rifampicin resistance meningococci.

## Methods

### Bacterial strains and bacterial proteins extraction

Two rifampicin resistant (RIF^R^) 870 and 901 strains and one rifampicin susceptible (RIF^S^) 1958 serogroup C meningococci were analysed. The resistant strains showed two already described [5] mutations in the *rpoB *gene, the Asp542Val and the His552Tyr. Strain 870 had caused fatal septicaemia in a 34 year-old man and strain 901, meningitis in a 1 year-old infant. The RIF^S ^1958 invasive strain was responsible for septicaemia in an infant aged 2, and since the absence of mutations in the *rpoB *gene, was chosen as control strain.

Bacterial protein extraction was performed according to the protocol previously described [13], with some modifications. In particular, the confluent bacterial growth was scraped from the plates and washed twice with PBS, suspended in 5 ml of lysis buffer (500 mM NaCl, 10 mM EDTA, 50 mM Tris pH 8.0) containing 0.3 mg/ml protease inhibitor (CompleteMini, Roche Diagnostic, Mannheim, Germany) and 150U DNase I (Roche Diagnostic).

The sample analysed by 2-DE approach corresponds to the cytosolic fraction, in which most of the proteins involved in the metabolic pathway and in essential biological processes have been described in bacteria.

### Two-dimensional gel electrophoresis

Before electrophoresis an aliquot of protein extract corresponding to 350 μg of each sample was precipitated by adding nine volumes of cold-ethanol and keeping at -20°C overnight. Samples were centrifuged at 14. 000 *g *for 15 min at 4°C and pellets were dried and then dissolved in 185 μl of a rehydration buffer containing 7 M urea, 2 M thiourea, 2% w/v CHAPS, 50 mM DTT, 0.2% v/v Bio-Lytes™pH range 3-10. Each sample was loaded on an 11-cm precast Immobiline strip with a linear pH 4-7 gradient and three replica maps were performed. First- and second-dimension electrophoresis, and image analysis were carried out as already described by Mignogna *et al. *[13].

### Protein identification

Spots selected according to the procedure previously described [13], were manually excised from gels and digested with trypsin. Digestion was performed at 37°C overnight. Briefly, after several destaining steps using 50 mM ammonium bicarbonate (15 min), 50% acetonitrile in 50 mM ammonium bicarbonate (10 min) and 100% acetonitrile (15 min), subsequently, about 100 ng of trypsin (Trypsin Gold, Mass Spectrometry Grade, Promega, Madison, WI, USA), solubilised in 10 μl of a 25 mM ammonium bicarbonate digestion buffer, were added to vacuum-dried gel.

An aliquot (1 μl) of each mixture peptide was mixed with the same volume of α-cyano-4-hydroxy-trans-cinnamic acid matrix solution (5 mg/ml) in 70% acetonitrile containing 0.1% TFA (v/v) for MALDI-ToF analysis, performed in a Voyager-DE STR instrument (Applied Biosystems, Framingham, MA) equipped with a 337 nm nitrogen laser and operating in reflector mode. Mass data were obtained by accumulating several spectra from laser shots with an accelerating voltage of 20 kV. Two tryptic autolytic peptides were used for the internal calibration (*m/z *842.5100 and 2807.3145).

Identification by peptide mass fingerprint (PMF), was performed using the Mascot search engine version 2.2 [14] against NCBlnr database (10386837 sequences). Up to one missed cleavage, 50 ppm measurement tolerance, oxidation at methionine (variable modification) and carbamidomethyl cysteine (fixed modification) were considered. Post-translational modifications were not taken into account. Identifications were validated when the probability-based Mowse protein score was significant according to Mascot [15].

### Statistical analysis of 2-DE maps

For gel comparison, a statistical approach was applied when determining differentially expressed proteins using the PDQuest software (version 7.2.0, BioRad). Student's *t*-test was performed with 90% significance level to determine which proteins were differentially expressed between the susceptible and resistant strain. Thresholds for assigning differential expression between the two pools were set at a minimum 2-fold change for up-regulation and 0.5-fold for down-regulation. This fold change threshold was chosen to obtain significant changes in protein expression. To minimize variation due to experimental factors, the intensity of each spot was normalized on the basis of the total integrated optical density for the examined gel.

### Sequence analysis of the genes encoding the four shifted proteins

Chromosomal DNAs were extracted by using the QIAamp DNA mini kit (Qiagen, Hilden, Germany) according to the manufacturer's instruction. The encoding genes for the four shifted proteins of the meningococcal isolates were amplified by PCR and sequenced with primers designed on conservative regions of corresponding genes from *N. meningitidis *FAM18 (NCBI accession number AM421808) (Table 1). All reactions were carried out with 100ng of purified chromosomal DNA, 5 μl of 10× reaction buffer, 0.01 mM of dNTP solution (Finnzymes, Finland), 2.5U HotStartTaq (Qiagen), 25pmol of each primer and sterile water to a final volume of 50 μl. Three different cycle conditions, changing for the annealing temperatures, were set up for the putative oxidoreductase, putative phosphate acyltransferase, putative zinc-binding alcohol dehydrogenase genes, respectively. In particular, 95°C for 15 minutes (hot-start); 30 cycles of 95°C for 30 seconds, 54°C -55°C-58°C for 30 seconds, and 72°C for 1 minute; and a final extension reaction at 72°C for 7 minutes.

**Table 1:** Primers for amplification and sequence analyses of genes encoding the four shifted proteins found in rifampicin resistant meningococci

Primer	Sequence (5'→3')	Protein encoded (Locus tag)
ADZ-f	^576170^GCGTTTCAGACGGCATTTGT^576189^*	putative zinc-binding alcohol dehydrogenase (NMC0547)
ADZ-r	^577320^GCCAGATTCAGACGGTATTCC^577300^*	
ICD-f	^893762^ACGACGAATGTTCAGACGG^893780^*	isocitrate dehydrogenase (NMC0897)
ICD-r	^896097^TGCCATAATAGCCACGCAC^896079^*	
PTA-f	^607259^AAGCCGTTTGTCAGCCTT ^607276^*	putative phosphate acyltransferase Pta (NMC0575)
PTA-r	^608401^CGGGCGTATTGGAAGGTTT ^608383^*	
POX-f	^445746^AAAGCCGGATAAGTGGGAAC^445765^*	putative oxidoreductase (NMC0426)

* the position referring to the corresponding accession number of *N. meningitidis *strain FAM18, accession number AM421808.

Cycle conditions for the isocitrate dehydrogenase gene were set at 95°C for 15 minutes (hot-start); 30 cycles of 95°C for 30 seconds, 58°C for 30 seconds, and 72°C for 2 minutes; a final extension reaction at 72°C for 7 minutes.

The PCR products were analysed using the BLAST program http://www.ncbi.nlm.nih.gov/BLAST/; amino acid sequences were aligned using the ClustalW program http://www.ebi.ac.uk/clustalw/.

### Analysis of *N. meningitidis *rifampicin resistant and susceptible strains growth curves

Meningococcal strains were incubated overnight on GC agar base (Oxoid, Basingstoke, UK) plates at 37°C with 5% CO_2. _Isolated colonies were inoculated in 4 ml GC broth plus rifampicin slightly stirring. The broth suspensions were immediately adjusted to an initial OD_600 _of 0.08 and the growth was measured by reading optical density (OD) every 60 min.

The RIF^R ^strains were grown on plates with 50 μg/ml of rifampicin. Each growth curve was repeated three times.

## Results

### Analysis of protein expression by 2-DE

**Figure 1:**
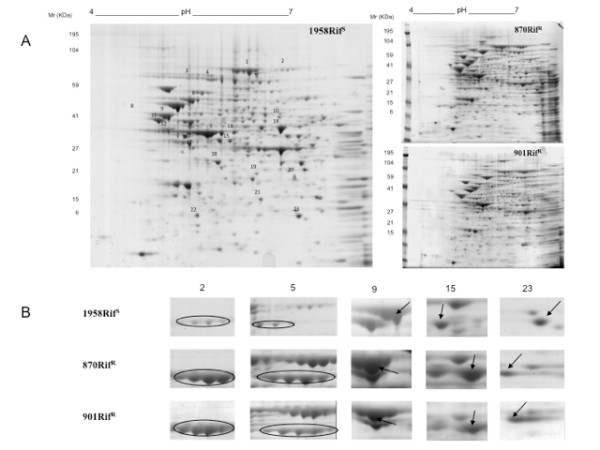
**2-DE of proteins corresponding to cytosolic fractions from (A) rifampicin resistant 870 and 901 and rifampicin susceptible 1958 *N. meningitidis *strains; (B) close-up views of some protein spots differentially expressed; spot numbers correspond to those reported in the panel A**.

The 2-DE gels were performed in three replicates and variations in spot intensity were confirmed by statistical analysis. Representative 2-DE maps of the two RIF^R ^870 and 901 strains and one RIF^S ^1958, are reported in figure 1A. The number of detected spots was in a range of 320 to 450 for all replicates.

As shown in figure 1A, there was a high similarity in protein pattern among the resistant and susceptible strains, with the majority of proteins ranging from pI 4 to 6 and with a molecular weight from 6000 to 195000 Da.

Protein identification by 2-DE gels and relative expression data were compared using PDQuest software; spots with a minimum of 2-fold change were chosen to define an up-expressed protein and 0.5-fold to define a down-expressed protein.

A total of twenty-three spots were found to be differentially expressed in both rifampicin resistant strains compared to the susceptible; all of them were subjected to the peptide mass fingerprinting (PMF) by MALDI-ToF analysis for protein identification. We performed the same analysis also on two isogenic rifampicin resistant meningococci mutants: the reference strain *N. meningitidis *serogroup B MC58 and one clinical isolate (data not shown).

Table 2 shows the functional classification of 23 up- and down-expressed proteins according to Universal Protein Knowledgebase (UniProtKB) database [16].

**Table 2:** List of the 23 differentially expressed proteins found in rifampicin resistant *Neisseria meningitidis *strains

Spot n	Protein name (gene) ^a^	Protein accession number	Ordered Locus Name^b^	Sequence coverage %	Mowse Score	MW_t_/pI_t_	Expression level ^c^	UniProtKB Functional classification ^d^
1	Aconitate hydratase (*acn*B)	A1KUZ6	NMC1492	51	403	93412/5.38	up	Carbohydrate metabolism: TCA cycle
2	Piruvate dehydrogenase subunit E1 (*ace*E)	A1KUG5	NMC1278	53	426	99915/5.60	up	Carbohydrate metabolism: pyruvate metabolism
3	Putative phosphoenolpyruvate synthase (*pps*A)	A1KSM6	NMC0561	26	165	87128/6.01	up	Carbohydrate metabolism: pyruvate metabolism
4	Elongation factor G (*fus*A)	A1KRH0	NMC0127	30	245	77338/5.08	up	Genetic Information Processing: protein synthesis
5	Isocitrate dehydrogenase (*icd*)	A1KTJ0	NMC0897	27	229	80313/5.53	up*	Carbohydrate metabolism: TCA cycle
6	60 kDa chaperonin (*gro*L)	A1KW52	NMC1948	41	206	57535/4.90	down	Genetic Information Processing: protein folding
7	ATP synthase subunit α (*atp*A)	A1KW13	NMC1908	62	281	55481/5.50	down	Energy metabolism: oxidative phosphorilation
8	N utilisation substance protein A (*nus*A)	A1KV50	NMC1556	71	426	55745/4.54	up	Genetic Information Processing: protein synthesis
9	Putative phosphate acyltransferase (NMC0575)	A1KSN9	NMC0575	47	263	57551/5.47	up*	Carbohydrate metabolism: propanoate metabolism
10	Probable malate:quinone oxidoreductase (*mqo*)	A1KWH2	NMC2076	36	178	54091/5.58	down	Carbohydrate metabolism: TCA cycle
11	Trigger factor (*tig*)	A1KUE0	NMC1250	51	209	48279/4.76	down	Genetic Information Processing: protein folding
12	Enolase (*eno*)	A1KUB6	NMC1220	25	129	46319/4.78	down	Carbohydrate metabolism: glycolysis
13	Cell division protein (*fts*A)	A1KVK9	NMC1738	40	132	44348/5.33	down	Genetic Information Processing: cell division
14	Glutamate dehydrogenase (*gdh*A)	A1KVB4	NMC1625	54	221	48731/5.80	up	Energy metabolism: amino acid metabolism
15	Putative zinc-binding alcohol dehydrogenase (NMC0547)	A1KSL2	NMC0547	38	235	38283/5.32	down*	Carbohydrate metabolism: butanoate metabolism
16	Succinyl-CoA ligase [ADP-forming] subunit beta (*suc*C)	A1KTM6	NMC0935	26	125	41567/5.01	up	Carbohydrate metabolism: TCA cycle
17	DNA-directed RNA polymerase subunit α (*rpo*A)	A1KRJ9	NMC0158	41	184	36168/4.94	up	Genetic Information Processing: transcription
18	Carboxyphosphonoenol pyruvate phosphonomutase (*prp*B)	A1KVK6	NMC1733	73	234	31876/5.22	down	Carbohydrate metabolism: propanoate metabolism
19	Putative malonyl Co-A acyl carrier protein transacylase (*fab*D)	A1KRY7	NMC0305	57	158	31958/5.44	down	Lipid metabolism: fatty acid biosynthesis
20	Septum site-determining protein (*min*D)	A1KRK2	NMC0161	29	143	29768/5.70	down	Genetic Information Processing: cell division
21	Putative two-component system regulator (NMC0537)	A1KSK4	NMC0537	74	181	24821/5.44	down	Environmental Information Processing: signal transduction
22	Peptidyl-prolyl cis-trans isomerase (*ppi*B)	A1KT50	NMC0744	84	260	18840/5.04	down	Genetic Information Processing: protein folding
23	Putative oxidoreductase (NMC0426)	A1KSA1	NMC0426	52	129	20759/5.74	down*	-

**^a ^**According to the UniProtKB/TrEMBL entry http://www.uniprot.org/.

**^b^**Ordered Locus Name in *Neisseria meningitidis *serogroup C/serotype 2a (strain ATCC 700532/FAM18)

**^c ^**Expression level of RIF ^R ^*versus *RIF ^S ^strains.

**^d ^**Functional classification of the proteins according to KEGG: Kyoto Encyclopedia of Genes and Genomes http://www.genome.jp/kegg/.

*Protein with changed pI in RIF ^R ^versus RIF ^S ^isolate.

Proteins belonging to the carbohydrate metabolism and the enzymes involved in the reactions of the tricarboxylic cycle (TCA) resulted up-expressed: in particular, the phosphenolpyruvate synthase [A1KSM6], the pyruvate dehydrogenase subunit E1 [A1KUG5], the glutamate dehydrogenase [A1KVB4], together with the isocitrate dehydrogenase [A1KTJ0], the succinyl-CoA synthetase subunit beta [A1KTM6] and the aconitate hydratase [A9M175]. Four proteins belonging to different metabolic pathways and those responsible for ATP production were down-expressed in both resistant strains: the malate quinone oxidoreductase [A1KWH2], the enolase [A1KUB6], the putative zinc-binding alcohol dehydrogenase [A1KSL2], the carboxyphosphonoenol pyruvate phosphonomutase [A9M2G6] and the F0F1 ATP synthase subunit α [A9M121 (Table 2).

A second group of proteins is involved in the regulation of the gene expression: the elongation factor G [A1KRH0], the transcription elongation factor NusA [C9WY90], and the DNA-directed RNA polymerase subunit α [A1KRJ9] were up-expressed. On the contrary, the DNA-binding response regulator [A9M2D6], involved in the transcription, the trigger factor [A1KUE0] involved in protein export, the 60 kDa chaperonin [A1KW52], that prevents misfolding and promotes the refolding of polypeptides, and the peptidyl-prolyl cis-trans isomerase [A9M3M5**]**, which accelerates the folding of proteins, were down-expressed.

The cell division protein [A1KVK9], the septum site-determining protein MinD [A9M3T7], the malonyl-CoA-acyl carrier protein transacylase [A1KRY7] and the putative oxidoreductase [A9M1W2], also resulted down-expressed.

Four of the 23 listed proteins in the Table2 had a different pI in both the resistant strains. The difference in the pI was well visualised in the 2-DE gels. As shown in figure 1B, the isocitrate dehydrogenase (spot 5) and the putative zinc-binding alcohol dehydrogenase (spot 15) were shifted to a more basic pI, while the putative phosphate acetyltransferase (spot 9) and the putative oxidoreductase (spot 23) were shifted to a more acidic pI.

### Sequence analysis of the genes encoding the shifted proteins

The four genes encoding proteins with a different pI were sequenced. In particular, NMC0426, NMC0547, NMC0575 and NMC0897 genes of the two resistant strains showed nucleotide mutations resulting in amino acid changes absent in the susceptible strain. All of them were considered missense mutations: 1) an arginine was replaced by a histidine residue at position 21 in the putative oxidoreductase; 2) an aspartic acid was replaced by a glycine residue at position 81 in the putative zinc-binding alcohol dehydrogenase; 3) an alanine was replaced by a serine residue at position 331 and an arginine by a leucine at position 351 in the putative phosphate acetyltransferase; 4) a threonine was replaced by an alanine residue at position 166; an isoleucine by a threonine at position 189; a glutamic acid by an alanine at position 212; an aspartic acid by a glycine at position 284; a valine by an alanine at position 710 and a valine by an alanine at position 731 in the isocitrate dehydrogenase.

### Analysis of bacteria growth curves

**Figure 2:**
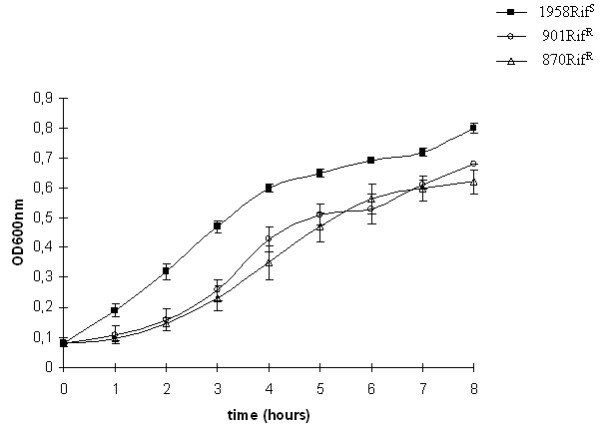
**Growth curves in GC broth of rifampicin resistant and susceptible strains**. Error bars represent the standard deviation of three culture replicates.

The rifampicin resistant strains grew normally and showed the same colony appearance as the rifampicin susceptible isolate, on GC agar plates with the naked eye or with a light microscope (data not shown). As shown in figure 2, after GC broth inoculation, there were differences in growth between the susceptible and resistant strains from the starting point of the inoculation (T0) to the stationary phase. In particular, the growth of the resistant strains showed primarily a delay in the onset of the logarithmic phase compared with the susceptible strain with different maximal OD_600 _= 0.82 of 1958, OD_600 _= 0.7 of 901, OD_600 _= 0.65 of 870 (figure 2).

## Discussion

As a transformable bacterium *Neisseria meningitidis *is incline to acquire exogenous bacterial DNAs, but it has been relatively slow to acquire resistance. However, since it is a severe disease it is very important to monitor changes in the level of antibiotic susceptibility among clinical isolates. Resistance to rifampicin is only occasionally observed but the isolation of a resistant strain poses serious problems in managing the prophylaxis of close contacts. At present, it is unknown how changes in resistant phenotype correspond to different protein expression profiles.

Some studies reveal that the molecular mechanism of resistance is correlated to different amino acid changes in a short central region of the *rpo*B gene encoding the β-subunit of the RNA polymerase [3,17]. Moreover, a scarce virulence of rifampicin resistant *N. meningitidis *isolates has been proved in an *in vivo *model [2].

It is interesting to focus on adaptation mechanisms under antibiotic challenge which have a cost in terms of fitness [18]. The results described in this paper permit to hypothesize that compensation for the rifampicin resistance phenotype may be responsible for the different protein expression in meningococcus. The phenomenon is not so rare among bacterial pathogens and the proteomic approach facilitates the comprehensive analysis of protein content. Most of the proteins recovered in the 2-DE maps belong to the cytosolic fraction. The latter permits to analyse differences in those proteins involved in metabolic pathways including the RNA polymerase, as the molecular target of rifampicin resistance. On the basis of the catalogue of proteins of the reference *N. meningitidis *strain MC58 [13], protein expression in two rifampicin resistant and one susceptible meningococci was analysed.

The proteomic approach allows us to define the differences in protein pathways compared to the protein set of rifampicin susceptible meningococci.

In our experimental conditions the soluble proteins obtained between pI 4 and 7 were identified in the different set of metabolic pathways. In particular, the results revealed a decrease of proteins, such as the 60 kDa chaperonin, trigger factor and peptidyl-prolyl cis-trans isomerase, involved in the accurate folding of polypeptides. Such results suggest that the bacteria may direct their metabolism towards the production of new polypeptide chains with a high energy cost.

Moreover, the proteins involved in crucial metabolic pathways showed an increased expression with particular regard to the catabolism of the pyruvate: the phosphoenolpyruvate synthase, involved in the conversion of pyruvate into phosphoenolpyruvate, and the pyruvate dehydrogenase subunit E1, that catalyzes the pyruvate decarboxylation into acetyl-CoA. Pyruvate is a key intersection in several metabolic pathways in bacteria [19], and so the altered expression of its catabolites may be reflected in the different pathways it generates. Three proteins, the putative phosphate acyltransferase, the carboxy phosphoenol pyruvate phosphomutase and the putative zinc-binding alcohol dehydrogenase, involved in the TCA cycle, gluconeogenesis and oxidation reaction, were differentially expressed. Similarly to the pyruvate, the acetyl-CoA too is an important molecule in the bacterial metabolism, since it is the starting point of many biochemical reactions [20]. Its main use is to convey the carbon atoms within the acetyl group to the TCA cycle to be oxidized for energy production. In this oxidative direction the two rifampicin resistant isolates showed an up-expression of the three main proteins of the TCA cycle: the aconitate hydratase, the isocitrate dehydrogenase and succinyl-CoA synthetase subunit beta. These results were in agreement with findings in a comparative study on resistant *Acinetobacter baumannii *[21].

The glutamate dehydrogenase, one of the essential enzymes for meningococcal pathogenesis in the infant rat model [22], was also up-regulated; this is of particular relevance since it belongs to the amino acid biosynthesis.

One of the advantages of the proteomic approach is that protein modifications that lead to changes in charge or size can directly be visualized [23]. In fact, four proteins in both resistant strains displayed a shift in their pI. The pI shifts were confirmed by the presence of amino acid changes due to missense mutations. In particular, the substitution of the cationic amino acid arginine with the neutral leucine was responsible for the acidic shift of putative phosphate acetyltransferase. On the other hand, the basic shift of putative zinc-binding alcohol dehydrogenase and isocitrate dehydrogenase was due to mutations of aspartic acid and glutamic acid to neutral ones. It is noteworthy that the two rifampicin resistant strains showed a different growth curve with a longer lag phase compared to the susceptible isolate. The analysis revealed that most differences in protein expression patterns were genetically encoded rather than induced by antibiotic exposure. Over-expression of stress proteins was expected, as they represent a common non-specific response by bacteria when stimulated by different shock conditions. Positive transcription regulators were found to be over-expressed in rifampicin resistance, suggesting that bacteria could activate compensatory mechanisms to assist the transcription process in the presence of RNA polymerase inhibitors. Other differences in expression profiles were related to proteins involved in central metabolism; these modifications suggest metabolic disadvantages of resistant mutants compared to sensitive ones. Of particular interest are the proteins involved in the cell division site. The altered proteins can affect the integrity of the Z ring at various stages. In the same way, it was hypothesized that the Z ring assembly could be both coordinated with the cell cycle and rendered responsive to cellular and environmental stresses.

The analysis of the protein differentially expressed may suggest the intricate series of events occurring in these strains. In this light, the growth results may be partially explained by a decrease expression of proteins such as the cell division protein and the septum site-determining protein MinD.

## Conclusions

Our findings reveal that we need a deeper understanding of the interplay between antibiotic resistance, biological fitness and virulence. Although our results are not sufficient to establish an unequivocal association between the differential protein expression and the resistant phenotype, they may be considered a starting point in understanding the decreased invasion capacity of

*N. meningitidis *rifampicin resistant strains. In fact, they support the hypothesis that the presence of more than one protein differentially expressed, having a role in the metabolism, influences the ability to infect and to spread in the population. Different reports have described and discussed how a drug resistant pathogen shows a high biological cost for survival [24,25] and that may also explain why, for some pathogens, the rate of resistant organisms is relatively low considering the widespread use of a particular drug. This seems the case of rifampicin resistant meningococci.

Only the combination gained from different experimental methods and clinical data reporting will enable to model the adaptation response of such strains in their physiological network. Our aim was to improve knowledge of the microbial physiology of resistant meningococci and understand why, despite widespread use of rifampicin in prophylactic treatment, the resistant isolates continue to be so rare.

## Authors' contributions

AN performed protein extractions from the strains and drafted the manuscript. CF characterized the strains. GM and AG performed the 2-DE and mass spectrometry experiments, the statistical analysis and helped in the manuscript revision. MES contributed the final 2-DE analysis. PS conceived the study, designed and supervised the work and edited the manuscript. All authors read and approved the final manuscript.

## Competing interests

The authors declare that they have no competing interests.

## Ethical approval

Not required.

## References

[B1] Rainbow J Cebelinski E Bartkus J Glennen A Boxrud D Lynfield R Rifampin-resistant meningococcal disease Emerg Infect Dis 2005 11 977 979 15963302 10.3201/eid1106.050143 PMC3367591

[B2] Taha MK Zarantonelli ML Ruckly C Giorgini D Alonso JM Rifampin-resistant *Neisseria meningitidis* Emerg Infect Dis 2006 12 859 860 16710955 10.3201/eid1205.051296 PMC3374432

[B3] Carter PE Abadi FJ Yakubu DE Pennington TH Molecular characterization of rifampin-resistant *Neisseria meningitidis* Antimicrob Agents Chemother 1994 38 1256 1261 8092823 10.1128/aac.38.6.1256 PMC188195

[B4] Nolte O Rifampicin resistance in *Neisseria meningitidis*: evidence from a study of sibling strains, description of new mutations and notes on population genetics J Antimicrob Chemother 1997 39 747 755 10.1093/jac/39.6.747 9222044

[B5] Stefanelli P Fazio C La Rosa G Marianelli C Muscillo M Mastrantonio P Rifampicin-resistant meningococci causing invasive disease: detection of point mutations in the *rpoB *gene and molecular characterization of the strains J Antimicrob Chemother 2001 47 219 222 10.1093/jac/47.2.219 11157912

[B6] Skoczynska A Ruckly C Hong E Taha MK Molecular characterization of resistance to rifampicin in clinical isolates of *Neisseria meningitidis* Clin Microbiol Infect 2009 15 1178 1181 10.1111/j.1469-0691.2009.02783.x 19456827

[B7] Abadi FJ Carter PE Cash P Pennington TH Rifampin resistance in *Neisseria meningitidis *due to alterations in membrane permeability Antimicrob Agents Chemother 1996 40 646 651 8851587 10.1128/aac.40.3.646 PMC163174

[B8] Pan W Spratt BG Regulation of the permeability of the gonococcal cell envelope by the *mtr *system Mol Microbiol 1994 11 769 775 10.1111/j.1365-2958.1994.tb00354.x 8196548

[B9] Hagman KE Pan W Spratt BG Balthazar JT Judd RC Shafer WM Resistance of *Neisseria gonorrhoeae *to antimicrobial hydrophobic agents is modulated by the *mtr*RCDE efflux system Microbiology 1995 141 Pt 3 611 622 10.1099/13500872-141-3-611 7711899

[B10] Rouquette-Loughlin CE Balthazar JT Hill SA Shafer WM Modulation of the *mtr*CDE-encoded efflux pump gene complex of *Neisseria meningitidis *due to a Correia element insertion sequence Mol Microbiol 2004 54 731 741 10.1111/j.1365-2958.2004.04299.x 15491363

[B11] Wang Q Yue J Zhang L Xu Y Chen J Zhang M Zhu B Wang H A newly identified 191A/C mutation in the Rv2629 gene that was significantly associated with rifampin resistance in *Mycobacterium tuberculosis* J Proteome Res 2007 6 4564 4571 10.1021/pr070242z 17970586

[B12] Bernardini G Braconi D Santucci A The analysis of *Neisseria meningitidis *proteomes: Reference maps and their applications Proteomics 2007 7 2933 2946 10.1002/pmic.200700094 17628027

[B13] Mignogna G Giorgi A Stefanelli P Neri A Colotti G Mara B Schinina ME Inventory of the proteins in *Neisseria meningitidis *serogroup B strain MC58 J Proteome Res 2005 4 1361 1370 10.1021/pr0500511 16083288

[B14] Perkins DN Pappin DJ Creasy DM Cottrell JS Probability-based protein identification by searching sequence databases using mass spectrometry data Electrophoresis 1999 20 3551 3567 10.1002/(SICI)1522-2683(19991201)20:18<3551::AID-ELPS3551>3.0.CO;2-2 10612281

[B15] Pappin DJ Peptide mass fingerprinting using MALDI-TOF mass spectrometry Methods Mol Biol 2003 211 211 219 12489433 10.1385/1-59259-342-9:211

[B16] Wu CH Apweiler R Bairoch A Natale DA Barker WC Boeckmann B Ferro S Gasteiger E Huang H Lopez Magrane M Martin MJ Mazumder R O'Donovan C Redaschi N Suzek B The Universal Protein Resource (UniProt): an expanding universe of protein information Nucleic Acids Res 2006 34 D187 D191 10.1093/nar/gkj161 16381842 PMC1347523

[B17] Nolte O Muller M Reitz S Ledig S Ehrhard I Sonntag HG Description of new mutations in the *rpoB *gene in rifampicin-resistant *Neisseria meningitidis *selected in vitro in a stepwise manner J Med Microbiol 2003 52 1077 1081 10.1099/jmm.0.05371-0 14614066

[B18] Andersson DI Levin BR The biological cost of antibiotic resistance Curr Opin Microbiol 1999 2 489 493 10.1016/S1369-5274(99)00005-3 10508723

[B19] Sauer U Eikmanns BJ The PEP-pyruvate-oxaloacetate node as the switch point for carbon flux distribution in bacteria FEMS Microbiol Rev 2005 29 765 794 10.1016/j.femsre.2004.11.002 16102602

[B20] El-Mansi M Cozzone AJ Shiloach J Eikmanns BJ Control of carbon flux through enzymes of central and intermediary metabolism during growth of *Escherichia coli *on acetate Curr Opin Microbiol 2006 9 173 179 10.1016/j.mib.2006.02.002 16530464

[B21] Fernandez-Reyes M Rodriguez-Falcon M Chiva C Pachon J Andreu D Rivas L The cost of resistance to colistin in *Acinetobacter baumannii*: a proteomic perspective Proteomics 2009 9 1632 1645 10.1002/pmic.200800434 19253303

[B22] Sun YH Bakshi S Chalmers R Tang CM Functional genomics of *Neisseria meningitidis *pathogenesis Nat Med 2000 6 1269 1273 10.1038/81380 11062540

[B23] Hecker M Antelmann H Buttner K Bernhardt J Gel-based proteomics of Gram-positive bacteria: a powerful tool to address physiological questions Proteomics 2008 8 4958 4975 10.1002/pmic.200800278 19003856

[B24] Andersson DI Persistence of antibiotic resistant bacteria Curr Opin Microbiol 2003 6 452 456 10.1016/j.mib.2003.09.001 14572536

[B25] Handel A Regoes RR Antia R The role of compensatory mutations in the emergence of drug resistance PLoS Comput Biol 2006 2 e137 10.1371/journal.pcbi.0020137 17040124 PMC1599768

